# Serum Alkaline Phosphatase Levels in Healthy Children and Evaluation of Alkaline Phosphatasez-scores in Different Types of Rickets

**DOI:** 10.4274/jcrpe.v3i1.02

**Published:** 2011-02-23

**Authors:** Serap Turan, Burcu Topcu, Ibrahim Gökçe, Tülay Güran, Zeynep Atay, Anjumanara Omar, Teoman Akçay, Abdullah Bereket

**Affiliations:** 1 Marmara University, Department of Pediatric Endocrinology, Istanbul, Turkey; 2 Marmara University, Department of Pediatrics, İstanbul, Turkey; 3 Şişli Etfal Education and Research Hospital, Department of Pediatric Endocrinology, Istanbul, Turkey; +90 216 327 10 10-616+90 216 325 15 89serap.turan@marmara.edu.trMarmara University Department of Pediatric Endocrinology, Tophanelioglu Cad. Altunizade, Istanbul, Turkey

**Keywords:** Alkaline phosphatase, children, reference values, rickets

## Abstract

**Objective:** Serum alkaline phosphatase (ALP) levels show great variation with age and sex in children and adolescents. Additionally, different buffers used even in the same method cause variable results. This detail is not usually taken into account in the evaluation. We aimed to study pediatric age- and sex-specific reference ranges for ALP by colorimetric assay using p-nitrophenyl phosphate as substrate and diethanolamine as buffer and also to compare the ALP levels in patients with different types of rickets.

**Methods:** 1741 healthy children and adolescents (904 girls) were included in the study for normative data. 77 different ALP measurements from 38 nutritional rickets (NR), 7 vitamin D-dependent rickets (VDDR) and 8 hypophosphatemic rickets (HR) patients were included.

**Results:** Reference values for ALP were constructed. ALP levels demonstrated a tetraphasic course with two peaks at infancy and puberty. There was no difference in ALP levels between boys and girls until puberty. However, higher ALP levels were noted at 10-11 years in girls (p=0.02) and at 12-13, 14-15, 16-17 years in boys (p<0.001). ALP levels start to decline after age 12 and 14 in girls and boys, respectively.  Serum ALP levels were highest in the VDDR group and lowest in the HR group (median z-score values in HR, VDDR and NR were 3.6, 10.4 and 6.5, respectively; p<0.001). Similarly, plasma parathormone(PTH)  levels ranged from highest to lowest in the VDDR, NR and HR groups (median values: 525, 237 and 98 pg/mL, respectively; p<0.001).

**Conclusions: **This normative data will provide a basis for better evaluation of ALP levels determined by the described method. Furthermore, use of z-scores gives a more precise assessment of changes in ALP levels in rickets and other bone disorders.

**Conflict of interest:**None declared.

## INTRODUCTION

Alkaline phosphatases (ALP) are a group of cell membrane metalloenzymes that catalyze the hydrolysis of phosphate esters in an alkaline environment, generating an organic radical and inorganic phosphate (1). ALP is expressed mainly in bone, liver, intestines, proximal convoluted tubules of the kidney, and in the placenta. ALP released from these tissues constitutes the total amount measured in the blood. Total ALP activity changes with age and bone fraction, varying from 77% to 89% in children and from 58% to 67% in adults (3,4,5).  In contrast to ALP isoenzymes, total serum ALP is widely used in routine biochemical tests and can be performed in almost all laboratories. Traditionally, total serum ALP activity has been used as a biochemical marker for bone formation to assess osteoblastic activity in primary hyperparathyroidism, rickets, osteomalacia and Paget’s disease. Since ALP is a marker for osteoblastic activity, growing children have higher levels than fully grown individuals. Highest levels of ALP are detected during the rapid growth phases of childhood such as infancy and puberty (2,6,7,8). While it is known that reference values of serum ALP are highly dependent on age and sex in childhood, most commercial assay kits used in hospital laboratories provide reference values for only adults and no specific references are given for pediatric ages. Additionally, the colorimetric method using p-nitrophenyl phosphate as substrate is the method used by most clinical laboratories, but the buffervaries between assays. 2-amino-2-methyl-1-propanol (AMP) is the buffer 

recommended by both the American Association for Clinical Chemistry (AACC) (9) and the International Federation of Clinical Chemistry (IFCC) (10); this buffer is also used in the method described by Bretaudiere et al (11). On the other hand, diethanolamine (DEA) is recommended by the Scandinavian Society for Clinical Chemistry (SSCC) (12). Higher values were obtained with the SSCC method (12), owing to the increased sensitivity resulting from the use of DEA buffer. The lower values with the AACC/IFCC method (9, 10) can be attributed to a lower concentration determined with the AMP buffer. Consequently, there are great variations in serum ALP levels assayed in different laboratories and in the interpretation of the results. Therefore, it is necessary to state the buffer used in the assay when reporting the ALP levels. This is particularly important for the pediatric age group, in which the upper limit of reference ranges may show an almost twofold variation.

In this study, we aimed to establish pediatric age- and sex-specific reference ranges for serum total ALP by the colorimetric method using p-nitrophenyl phosphate as substrate and diethanolamine as buffer and run by Roche/Hitachi 917/MOD system. Additionally, we compared the ALP z-scores of patients with different types of rickets by using these reference ranges.

## METHODS

**Sample Selection**

We studied 1922 children aged 0 to 18 years who presented to the outpatient clinic for routine check-ups or for minor conditions requiring analysis of blood samples for diagnosis. Children who showed clinical signs of any acute or chronic illnesses affecting serum ALP levels were not included in the study. Children and adolescents under treatment with corticosteroids or anticonvulsants, those with neuromuscular disorders or movement impairment, and those with genetic syndromes or major congenital malformations were also excluded. Additionally, serum parathormone (PTH) levels were measured in children with ALP levels in the higher quartile according to their age groups and the children with high PTH levels were excluded from the study. 

The Ethics Committee of the Medical Faculty at the Marmara University approved the study. Informed consent was obtained from the parents of each child and from the child if older than 16 years of age. The study was performed between March 2003 and April 2006.

1741 children (904 girls and 837 boys) were included in the final analysis after excluding subjects who were found to have clinical or laboratory evidence of conditions, which might affect the serum ALP levels. The age- and sex-specific reference ranges for serum ALP levels were constructed from the data of these 1741 children.  

Additionally, serum ALP levels of 77 different samples from 53 patients with active rickets were analyzed to determine the ALP z-scores in different types of rickets and validate the constructed reference data. This group included 39 samples from 38 children with nutritional rickets (NR) (27 males and 11 females), 19 samples from 7 (3 males, 4 females) with vitamin D-dependent rickets (VDDR) and 19 samples from 8 (3 males, 5 females) with hypophosphatemic rickets (HR). 

**Serum Analyses**

Blood samples were drawn from an antecubital vein between 08.00-13.00 hours and centrifuged thereafter. Blood samples were transferred to the central laboratory for determination of ALP levels on the same day. Colorimetric assay with a standardized method usingp-nitrophenyl 

phosphate as substrate and diethanolamine as buffer was run by Roche/Hitachi 917/MOD system. Detection limits of the assay were 5 to 2 000 U/L and higher activities were re-run after dilution.  The intra-assay coefficient of variation (CV) was 0.5%, whereas the interassay CV was 2.2%.

**Statistics**

Results were presented as the mean and standard deviation (SD) values in 11 different age groups as shown in Table 1.  Square root (SQR) transformation was used in the calculations since this transformation gave the best approximation to a normal distribution of the data. 

ALP z-score calculations were performed according to the following formula:

z-score = (SQR of ALP value- mean of SQR ALP values for the age group ) / SD of  SQR  ALP values for the age group

Statistical analyses were performed using Jandel SigmaStat version 2.0. The unpaired t-test was used to compare ALP levels between girls and boys in different age groups. Kruskal-Wallis one-way analysis of variance by ranks was used to compare ALP z-scores, PTH, calcium and phosphate leves between the different types of rickets.  

**Table 1:**
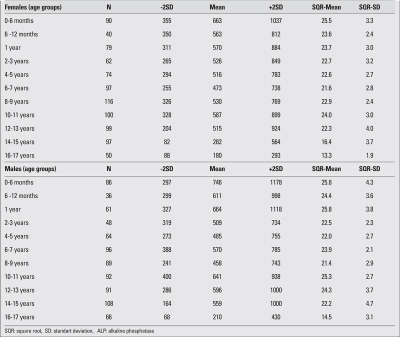
Mean serum ALP levels (U/L), -2SD, +2SD and square root transformed mean and SD values by age groups

## RESULTS

Serum ALP levels were highest in the first 6 months of life, decreased to relatively steady levels thereafter and started to increase again after age  9 years  (Figure 1). The pubertal peak levels were not as high as the levels detected in the first 6 months of life.  ALP levels (+2SD) exceeding 900 U/L were observed in two age groups in girls, namely 0-6 months and 12-13 years. In boys, these high levels were observed in six age groups, namely 0-6 months, 6-12 months, 1 year , 10-11 years, 12-13 years and 14-15 years Table 1. Serum ALP levels were similar in boys and girls until age 10 years, but higher ALP levels were noted at ages 10-11 years in girls (median values were 572 and 525, respectively; p= 0.02) and in three age groups, namely 12-13, 14-15 and 16-17 years in boys (median values were 630 U/L, 518 U/L, 587 U/L, 251 U/L, 369U/L and 167 U/L, respectively; p<0.001). ALP levels start to decline after 12 years of age in girls and after 14 years of age in boys, with upper ranges approaching the upper ranges of adults (240 U/L for females, 270 U/L for males) in girls (293 U/L), but not in boys (430 U/L) until they reach the ages of 16 to 18 years.  

**Serum ALP Levels in Children with Rickets**

**Figure 2:**
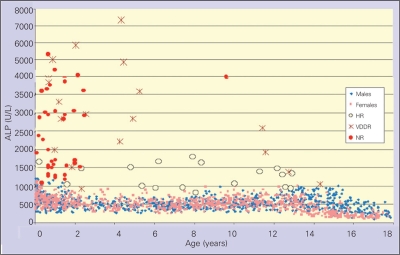
ALP levels in different types of rickets, full circles representing NR, stars VDDR and empty circles HR ALP: alkaline phosphatase, NR: nutritional rickets, VDDR: vitamin-D dependent ricket, HR: hypophosphatemic rickets

The serum ALP levels from patients with NR prior to treatment were analyzed according to normative data obtained in the study. Additionally, repeated measurements were 

performed in the groups of VDDR and HR before treatment as well as after the start of treatment if they continued to have signs of active rickets related to compliance or other problems at follow-up.  A total of 39 samples from 38 cases with NR, 19 samples from 7 cases with VDDR, and 20 samples from 8 cases with HR were obtained for final analysis. The biochemical data of patients with rickets are presented in Table 2. Serum ALP levels were highest in the VDDR group and lowest in the HR group. ALP z-score was significantly lower in the HR group (median: 3.6) than in the VDDR (median: 10.4) and NR (median: 6.5) groups (p<0.001). A similar pattern was observed for plasma PTH levels from highest to lowest in VDDR, NR and HR (median values of 525, 237 and 98 pg/mL, respectively). There were statistically significant differences among the three groups (p<0.001). Serum calcium levels were highest and serum phosphate levels were lowest in the HR group (HR vs. VDDR and NR; medians: 9.4 vs. 8.9 and 8.8 mg/dL for Ca, 2.5 vs. 2.9 and 2.9 mg/dL for phosphate, respectively; p<0.05). Serum ALP levels of the individual patients are shown in Figure 2. 

**(Figure 1):**
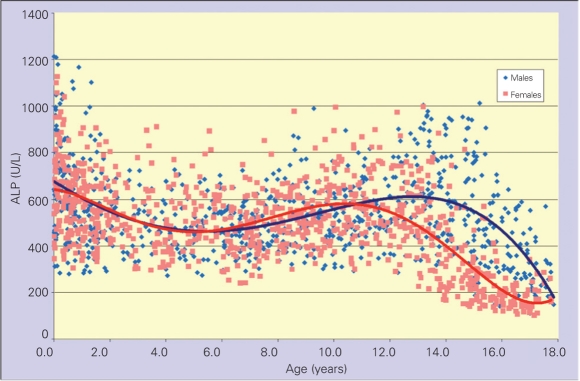
Serum ALP levels according to age showing a tetraphasic course from birth to adulthood ALP: alkaline phosphatase

**Table 1:**
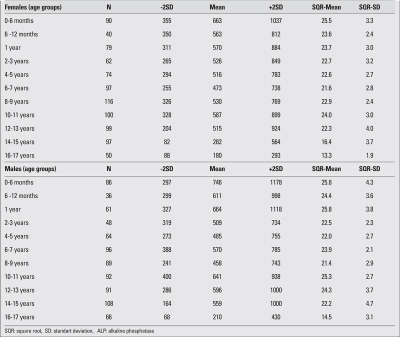
Mean serum ALP levels (U/L), -2SD, +2SD and square root transformed mean and SD values by age groups

**Table 2:**
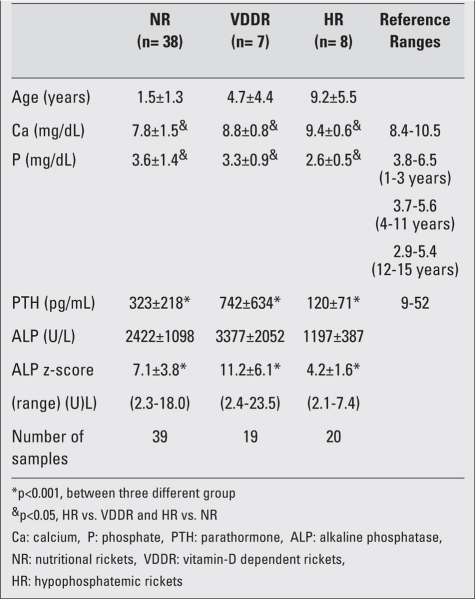
Serum calcium, phosphate, plasma parathyroid hormone levels with serum ALP levels and ALP z-scores in patients with different types of rickets. Mean±SD values are given.

## DISCUSSION

In the present study, we described age- and sex-specific serum ALP levels for ages 0 to 18 years. Serum ALP levels were determined by a widely used colorimetric assay, with p-nitrophenyl phosphate as substrate and diethanolamine as buffer. Serum ALP levels were also performed in patients with different types of rickets and were compared with the normative data.

In healthy children, normal ALP levels showed a tetraphasic pattern with highest levels in infancy and puberty and troughs at mid-childhood and at the end of puberty. ALP levels were similar during childhood in both sexes, but showed a dimorphic pattern after the age of 10. Studies evaluating bone-specific ALP levels demonstrate that 77% to 89% of the total ALP levels in children are of bone origin. The studies also indicate that the increase in ALP in pubertal ages reflects the rapid growth in puberty (3,4,5,7,13). Although, we did not check the pubertal status of the children in this study, we can clearly see the pubertal effect through the differences between boys and girls during the pubertal ages. As a result of earlier onset, earlier growth spurt and earlier completion of puberty, most girls in the 16-18 years age group reached ALP levels that were compatible with the upper ranges of adult females. Boys appear to reach the adult levels at later ages. These results confirm the previous data emphasizing that serum ALP levels decline to the adult ranges after the age of 20 in boys and ages 17-18 years in girls (2,7).

The mean ALP levels obtained in this study were similar to the references, in which DEA is used as a buffer (6,7,8), but higher than the values in the references using AMP as buffer (2,14). Different normative data according to the methodology necessitate development and use of z-scores when reporting ALP levels in clinical studies and case reports. This important issue is usually neglected. Thus, we also analyzed serum ALP levels in 3 different types of rickets and, to our knowledge, reported for the first time ALP z-scores for three different types of rickets patients. The highest ALP levels were detected in patients with VDDR and the lowest ones in HR patients; this  attern was valid also for PTH levels. Serum calcium levels were highest and, as expected, serum phosphate levels were lowest in HR patients. Furthermore, it is not possible to compare our data with ALP levels reported in the literature since there is no mention of buffers used for ALP measurement in rickets patients in most reports. We believe that using ALP z-score in the follow-up of patients, instead of using absolute ALP levels, would be more logical and free of age- and sex-related physiological changes in ALP levels and will give a better idea about the pathological process.

Additionally, not only normative upper limits of ALP, but also the lower ones are of interest in clinical settings, especially in hypophosphatasia patients. Hypophosphatasia is characterized by low serum ALP activity (hypophosphatasemia) due to loss-of-function mutation within TNSALP, the gene that encodes "tissue-nonspecific" ALP (15,16,17).

We hope that this normative data for ALP levels will be useful in establishing the diagnosis and monitoring the treatment in patients with abnormal ALP levels. Determination of z-scores for ALP will allow more precise assessment of changes in ALP levels in rickets and other bone disorders.

## References

[1] Warnes TW (1972). Alkaline phosphatase.. Gut.

[2] Schiele F, Henny J, Hitz J, Petitclerc C, Gueguen R, Siest G (1983). Total bone and liver alkaline phosphatases in plasma: biological variations and reference limits.. Clin Chem.

[3] Kruse K, Bartels H, Gunther H (1977). Serum alkaline phosphatase isoenzyme in childhood.. Eur J Pediatr.

[4] Statland BE, Nishi HH, Young DS (1972). Serum alkaline phosphatase:Total activity and isoenzyme determinations made by centrifugal fast analyzer.. Clin Chem.

[5] Plomteux G, Reginster N (1980). Measurement of the hepatic, intestinal and bony fractions of the serum alkaline phosphatase.. Ann Biol Clin (Paris).

[6] Crofton PM (1992). Wheat-germ lectin affinity electrophoresis for alkaline phosphatase isoforms in children: age-dependent reference ranges and changes in liver and bone disease.. Clin Chem.

[7] Fleisher GA, Eickelberg ES, Elveback LR (1977). Alkaline phosphatase activity in the plasma of children and adolescents.. Clin Chem.

[8] Penttilä IM, Jokela HA, Viitala AJ, Heikkinen E, Nummi S, Pystynen P, Saastamoinen J (1975). Activities of aspartate and alanine aminotransferases and alkaline phosphatase in sera of healthy subjects.. Scand J Clin Lab Invest.

[9] Tietz NW, Burtis CA, Duncan P, Ervin K, Petitclerc CJ, Rinker AD, Shuey D, Zygowicz ER. (1983). A reference method for measurement of alkaline phosphatase activity in human serum.. Clin Chem.

[10] International Federation of Clinical Chemistry (1983). Expert panel on enzymes, Part 5, IFCC method for alkaline  phosphat. Ase (orthophosphoric-monoester phosphohydrolase, alkaline optimum, EC 3.1.3.1).. Clin Chim Acta.

[11] Bretaudiere JP, Vassault A, Amsellem L, Pourci ML, Thieu-Phung H, Bailly M (1977). Criteria for establishing a standardized method for determining alkaline phosphatase activity in human serum.. Clin Chem.

[12] Committee on Enzymes of the Scandinavian Society for Clinical Chemistry and Clinical Physiology (1974). Recommended methods for the determination of four enzymes in blood.. Scand J Chin Lab Invest.

[13] Léger J, Mercat I, Alberti C, Chevenne D, Armoogum P, Tichet J, Czernichow P (2007). The relationship between the GH/IGF-I axis and serum markers of bone turnover metabolism in healthy children.. Eur J Endocrinol.

[14] Lockitch G, Halstead AC, Albersheim S, MacCallum C, Quigley G (1988). Age- and sex-specific pediatric reference intervals for biochemistry analytes as measured with the Ektachem-700 analyzer.. Clin Chem.

[15] Girschick HJ, Schneider P, Kruse K, Huppertz HI (1999). Bone metabolism and bone mineral density in childhood hypophosphatasia. Bone.

[16] Moulin P, Vaysse F, Bieth E, Mornet E, Gennero I, Dalicieux-Laurencin S, Baunin C, Tauber MT, De Gauzy JS, Salles JP (2009). Hypophosphatasia may lead to bone fragility: don't miss it.. Eur J Pediatr.

[17] Weiss MJ, Cole DE, Ray K, Whyte MP, Lafferty MA, Mulivor RA, Harris H (1988). A missense mutation in the human liver/bone/kidney alkaline phosphatase gene causing a lethal form of hypophosphatasia.. Proc Natl Acad Sci U S A.

